# Nutrient dependent *miR-184* aids in the survival of *Drosophila* larvae during low food conditions

**DOI:** 10.17912/micropub.biology.000541

**Published:** 2022-03-17

**Authors:** Jervis Fernandes, Elwina Thomas, Jishy Varghese

**Affiliations:** 1 School of Biology, Indian Institute of Science Education and Research Thiruvananthapuram (IISER TVM), Thiruvananthapuram, Kerala, India 695551

## Abstract

Nutrition is one of the critical factors known to regulate the development and growth of organisms. Lack of nutrients affects the proper functioning and survival of organisms. However, fluctuation of the levels of nutrients is quite common in a natural environment, and organisms have evolved various molecular and physiological means by which they can survive such conditions. microRNAs are short non-coding RNAs that play significant biological functions, primarily by acting as post-transcriptional buffers of noisy gene expression. Recent studies show that miR-184, a conserved microRNA, is expressed at higher levels in low nutrition conditions. Our experiments show that *miR-184* mutants showed enhanced lethality when raised in low nutrient food conditions. Here, we demonstrate the role of miR-184, a microRNA regulated by nutritional status, also helps in the survival of the larvae to adulthood in low food conditions.

**Figure 1. miR-184 levels are influenced by larval nutrient status and miR-184 plays a key role in larval survival during low nutrient conditions:**
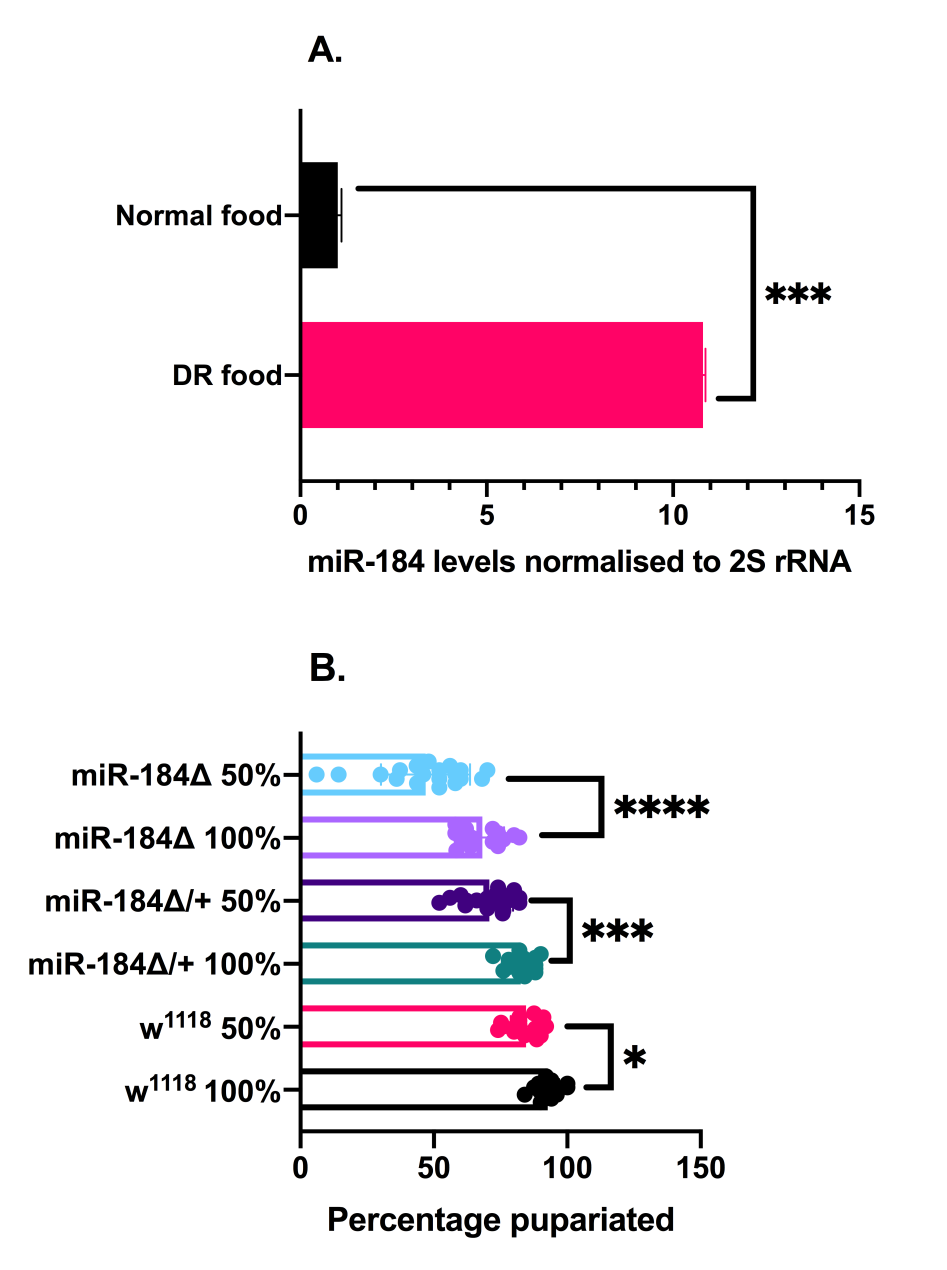
A. Expression of miR-184 in early L3 larvae reared in Normal food vs DR food (n=5). B. Percentage of larvae pupariated (n≥16). In A, an unpaired t-test was carried out to determine statistical significance. In B, a mixed-model ANOVA was carried out to determine statistical significance. Error bars represent standard deviation. n= number of biological replicates, * p < 0.05, ***p<0.001, ****p<0.0001.

## Description

Many different environmental and genetic factors regulate the growth and development of an organism. Nutrition, one of the key factors known to regulate development, does so by inducing epigenetic changes and modulating various molecular mechanisms, for instance, by regulating DNA methylation status (Kadayifci FZ et al. 2018) and modulating the activity of both Insulin and TOR signalling pathways (Grewal SS, 2009).

Appropriate levels of nutrients are essential for the normal metabolism, growth, and development of *Drosophila* larvae (Koyama T et al. 2020). Recently, the role of short noncoding RNAs called microRNAs in regulating growth and metabolism in *Drosophila* has been reported. With miR-277 observed to control branched-chain catabolism and regulate lifespan (​Esslinger SM et al. 2013​). miR-33 was demonstrated to regulate TAG metabolism in mammals and *Drosophila* (Clerbaux LA et al. 2021). *miR-278* mutants have excess circulating sugar levels and have low lipid stores, and they were also found to be insulin resistant (Teleman et al. 2006). miR-14 regulates insulin signalling in flies and thereby fat metabolism (Varghese J et al. 2010). miR-8 in *Drosophila* acts in the fatbody to promote insulin signaling and body growth (Hyun et al. 2009). A recent study by Gendron et al. identified let-7, miR-184, miR-34, and miR-8 as differentially expressed in varying dietary conditions in adult flies (​Gendron CM and Pletcher SD, 2017​). This work demonstrated the possible role these microRNAs could play in regulating the internal energy status following the external nutritional cues. Further, overexpression of miR-184 was found to decrease lifespan regardless of the food condition without any effects on triglyceride levels (​Gendron CM and Pletcher SD, 2017​). However, the possible roles of miR-184 during a low nutrient environment have not been tested yet.

miR-184 was also recently identified to increase in response to fasting in pancreatic islet cells (Tattikota SG et al. 2015). miR-184 was shown to be negatively regulated in a sucrose-rich diet, and the levels of this microRNA increased in response to starvation in the adult fly head (Tattikota SG et al. 2015). miR-184 was recently identified as an inhibitor of insulin secretion in pancreatic β-cell lines (​Morita S et al. 2013​). However, no effects on growth were observed when miR-184 was over-expressed in the *Drosophila* insulin-producing cells (​Suh YS et al. 2015​). miR-184 is a maternally deposited microRNA (Iovino N et al. 2009), expressed in a highly dynamic pattern throughout fly development with peak expression at stages 12–13 of embryogenesis and L3 larvae (Li P et al. 2011). It is known to play a role in various developmental processes and regulate many genes involved in the formation of septate junctions.

To check if miR-184 levels are affected in larvae raised on a low nutrient diet, we raised the larvae in food containing 50% diluted fly media (will be denoted as dietary restricted/DR food) in comparison to the standard food conditions in the lab (​Rehman N and Varghese J, 2021​). The level of the microRNA was measured and compared between early L3 larvae raised on standard food and larvae raised on DR food. The level of miR-184 was higher in larvae raised on DR food than those fed on standard food (Fig. 1A). Thus, upregulation of miR-184 in response to a low nutrient diet is observed even during larval growth stages. Homozygous deletion mutants of miR-184 are viable, with 79% of the larvae pupating and surviving till adulthood in normal food conditions (​Chen YW et al. 2014​). We found a significant increase in lethality in the *miR-184* homozygous mutant larvae compared to the heterozygous control larvae when reared on DR food (Fig. 1B). There was an increase in lethality when *miR-184* mutant homozygous larvae were compared to *w[1118]* larvae also, which was used as an additional control, when reared on DR food (Fig. 1B). No lethality was observed during the pupal stages, as all the pupae emerged as adult flies.

While lack of miR-184 during larval stages affected the survival of normally fed larvae, during low food conditions, the lack of miR-184 causes a further reduction of larval survival. In this context, an increase in miR-184 levels observed in response to diet restriction suggests an active role for miR-184 in circumventing the deleterious effects of nutritional stress. Thus, from our data, we conclude that miR-184 is important in the survival and development of *Drosophila* larvae during low food conditions. Although the molecular mechanism via which miR-184 fine-tunes development in accordance with the nutritional cues remains to be elucidated.

## Methods

**Food Composition:** The food preparation was according to Rehman and Varghese. 2021.

**microRNA qRT-PCR:** Total RNA was extracted from early L3 larvae using Trizol extraction method. miR-184 and 2SrRNA were reverse transcribed using a gene-specific stem-loop primer and the Takara MMLV reverse transcriptase. Followed by qRT-PCR with the use of TbGreen with the use of a Universal reverse primer and a miR-184 specific forward primer. The microRNA level was normalised to the levels of 2S rRNA.

**Survival to pupae:** Embryo collection cages were set up using apple juice agar plates and the flies were provided with yeast to stimulate egg laying. *miR-184Δ/CyO-GFP* flies were crossed with each other to obtain *miR-184Δ* homozygotes, non-GFP 1st instar larvae were collected, using a Leica fluorescence Stereoscope. *miR-184Δ/CyO-GFP* was crossed to *w[1118]* to obtain *miR-184Δ/+* heterozygotes, non-GFP 1st instar larvae were collected. *w[1118]* was used as an additional control. 50 L1 larvae were collected per vial and transferred to either of the food conditions (Normal food or DR food/50% food). Multiple independent collections were performed. The number of larvae pupating was noted in all the genotypes in different food conditions.

**Fly stocks:** *w[1118]* was a gift from Dr. Stephen Cohen’s lab. *miR-184* deletion mutant was purchased from the Kyoto stock center.

## Reagents

**Table:** 

#	Primer name	Sequence
1.	2S rRNA Forward primer	CATTCATGCTTGGACTACATATGGTTGAGG
2.	2S rRNA Stem loop primer	GTCGTATCCAGTGCAGGGTCCGAGGTATTCGCACTGGATACGTACAAC
3.	Universal Reverse primer	CCAGTGCAGGGTCCGAGGTA
4.	miR-190 Forward primer	GGGCGGCGAGATATGTTTGATATTCT
5.	miR-190 Stem loop primer	GTCGTATCCAGTGCAGGGTCCGAGGTATTCGCACTGGATACGACCAACCAAGAAT

**Table:** 

#	Stock line	DGRC Number	Genotype
1.	*miR-184 [Δ]*	116326	w[*]; TI{w[+mW.hs]=TI}miR-184[Δ]/CyO, P{w[+mC]=GAL4-twi.G}2.2, P{UAS-2xEGFP}AH2.2
